# Pioglitazone and PPAR-γ modulating treatment in hypertensive and type 2 diabetic patients after ischemic stroke: a national cohort study

**DOI:** 10.1186/s12933-019-0979-x

**Published:** 2020-01-07

**Authors:** Chi-Hung Liu, Tsong-Hai Lee, Yu-Sheng Lin, Pi-Shan Sung, Yi-Chia Wei, Yan-Rong Li

**Affiliations:** 1 Stroke Center and Department of Neurology, Linkou Chang Gung Memorial Hospital, Taoyuan, Taiwan; 2 College of Medicine, Chang Gung University, Taoyuan, Taiwan; 3 Division of Cardiology, Department of Internal Medicine, Chiayi Chang Gung Memorial Hospital, Chiayi, Taiwan; 4 Department of Medicine, College of Medicine, Chang Gung University, Taoyuan, Taiwan; 5 Department of Neurology, National Cheng Kung University Hospital, College of Medicine, National Cheng Kung University, Tainan, Taiwan; 6 Department of Neurology, Keelung Chang Gung Memorial Hospital, Keelung, Taiwan; 7 Institute of Neuroscience, National Yang-Ming University, Taipei, Taiwan; 8 Division of Endocrinology and Metabolism, Department of Internal Medicine, Linkou Chang Gung Memorial Hospital, and College of Medicine, Chang Gung University, No. 5, Fu-Hsing St, Kueishan, Taoyuan, 33333 Taiwan

**Keywords:** Diabetes mellitus, Hypertension, Ischemic stroke, Pioglitazone, PPAR-γ, Telmisartan

## Abstract

**Background and aim:**

Peroxisome proliferator-activated receptor-γ (PPAR-γ) modulating treatment may have cardiovascular benefits in type 2 diabetes mellitus (T2DM) patients after ischemic stroke (IS). However, whether there are additional benefits from intensive PPAR-γ modulating treatments in Asian patients with T2DM and hypertension (HTN) after IS remains unknown.

**Methods:**

Between 2001 and 2013, patients admitted due to IS were identified from the National Health Insurance Research Database of Taiwan. Patients with T2DM and HTN using angiotensin receptor blockers were further included. Eligible patients were divided into two groups: (1) pioglitazone and (2) non-pioglitazone oral anti-diabetic agent groups. Propensity score matching (1:2) was used to balance the distribution of baseline characteristics, stroke severity and medications. The primary outcome was recurrent IS. Subgroup analysis for recurrent IS in pioglitazone and/or telmisartan users, the trend of IS risks across different PPAR-γ intensity treatments, and dose-dependent outcomes across different pioglitazone possession ratios were further studied. Statistical significance was set at *p *< 0.05 and *p *< 0.1 for clinical outcomes and interaction of subgroup analyses, respectively.

**Results:**

There were 3190 and 32,645 patients in the pioglitazone and non-pioglitazone groups. Patients of the pioglitazone group had a lower risk of recurrent IS (subdistribution hazard ratio, 0.91; 95% confidence interval 0.84–0.99). Pioglitazone was also associated with reduced recurrent IS in patients who also used telmisartan (*p* for interaction = 0.071). A graded correlation was found a borderline significant trend between the intensity of PPAR-γ therapy and following IS (*p* = 0.076). The dose-dependent outcome also showed that a borderline significant trend that higher pioglitazone possession ratio was associated with a lower risk of recurrent IS (*p* = 0.068).

**Conclusions:**

The current study suggests that the use of pioglitazone in type 2 diabetic and hypertensive IS patients is associated with fewer recurrent IS events in an Asian population. Concurrent telmisartan use or a higher pioglitazone possession ratio may have a trend of increased pleiotropic effects, which could possibly be related to higher PPAR-γ effects. Future studies are warranted to confirm or refute the clinical effects and the possible mechanism of more intensive PPAR-γ-modulating treatments.

## Background

Stroke is one of the most common causes of death and disability worldwide, and risk factor modification is crucial for the prevention of ischemic stroke (IS). Among the well-established modifiable risk factors, diabetes mellitus (DM) and insulin resistance (IR) are independent risk factors for worse clinical outcomes of acute IS [1, 2], and long-term stroke recurrence [3]. IR may promote platelet aggregation, accelerate atherosclerosis, impair cerebrovascular reserve function, and cause endothelial dysfunction [3]. Therefore, patients with IR often have higher incidence rates of vascular events, including recurrent IS [3, 4]. Peroxisome proliferator-activated receptor-γ (PPAR-γ) is a known essential mediator for the maintenance of whole body insulin sensitivity [5]. Biochemical and metabolic regulators of PPAR-γ have been considered as therapeutic targets in cardiovascular diseases [6]. A previous Cochrane review demonstrated that PPAR-γ agonists may improve insulin sensitivity and probably reduce recurrent strokes and total cardiovascular death events [4].

Pioglitazone, which is a member of the thiazolidinedione class of drugs, is a potent PPAR-γ agonist and theoretically has protective vascular effects due to its mechanism of action [7, 8]. However, pioglitazone did not show any benefit compared to sulfonylurea in a low cardiovascular risk population in the ‘Thiazolidinediones or Sulphonylureas and Cardiovascular Accidents Intervention Trial’ [9]. For high cardiovascular risk patients, the occurrence of composite cardiovascular outcomes was lower in pioglitazone users in the ‘Insulin Resistance Intervention after Stroke’ (IRIS) study [8]. The results of meta-analyses suggested that pioglitazone could reduce the risk of stroke in IS patients with IR or DM [7, 8, 10, 11], but a meta-analysis study may have limitations due to the diversity of the study populations. Moreover, there are relatively limited data focusing on Asian patients from clinical trials. A Korea nested case–control study using real-world data demonstrated a benefit of pioglitazone on composite cardiovascular outcomes but not on recurrent IS in patients after acute IS [12]. It would be valuable to examine the benefits of pioglitazone in high cardiovascular risk Asian patients.

Angiotensin receptor blockers (ARBs) are antihypertensive drugs, which preferentially inhibit angiotensin type 1 receptors and therefore have several pleiotropic functions beyond their primary blood-pressure-lowering effects [13]. Among ARBs, telmisartan relatively yields higher PPAR-γ modulating activity and improves IR [13–17]. The safety of combining multiple PPAR-γ modulating drugs, such as pioglitazone and telmisartan, has been previously examined in animal models [18, 19]. Whether the combination results in better vascular protective effects for high-risk patients is an issue worth further investigation [13]. As to date, the answer has remained elusive [17]. This real-world study firstly examined the long-term outcomes of pioglitazone in Asian type 2 DM (T2DM) patients with hypertension (HTN) after IS. We further investigated whether intensive PPAR-γ modulating treatment, via a combination of pioglitazone and telmisartan, was associated with additional benefits beyond the target control of blood sugar and blood pressure (BP) levels in patients with T2DM and HTN.

## Methods

### Data source and patient identification

**Fig. 1:**
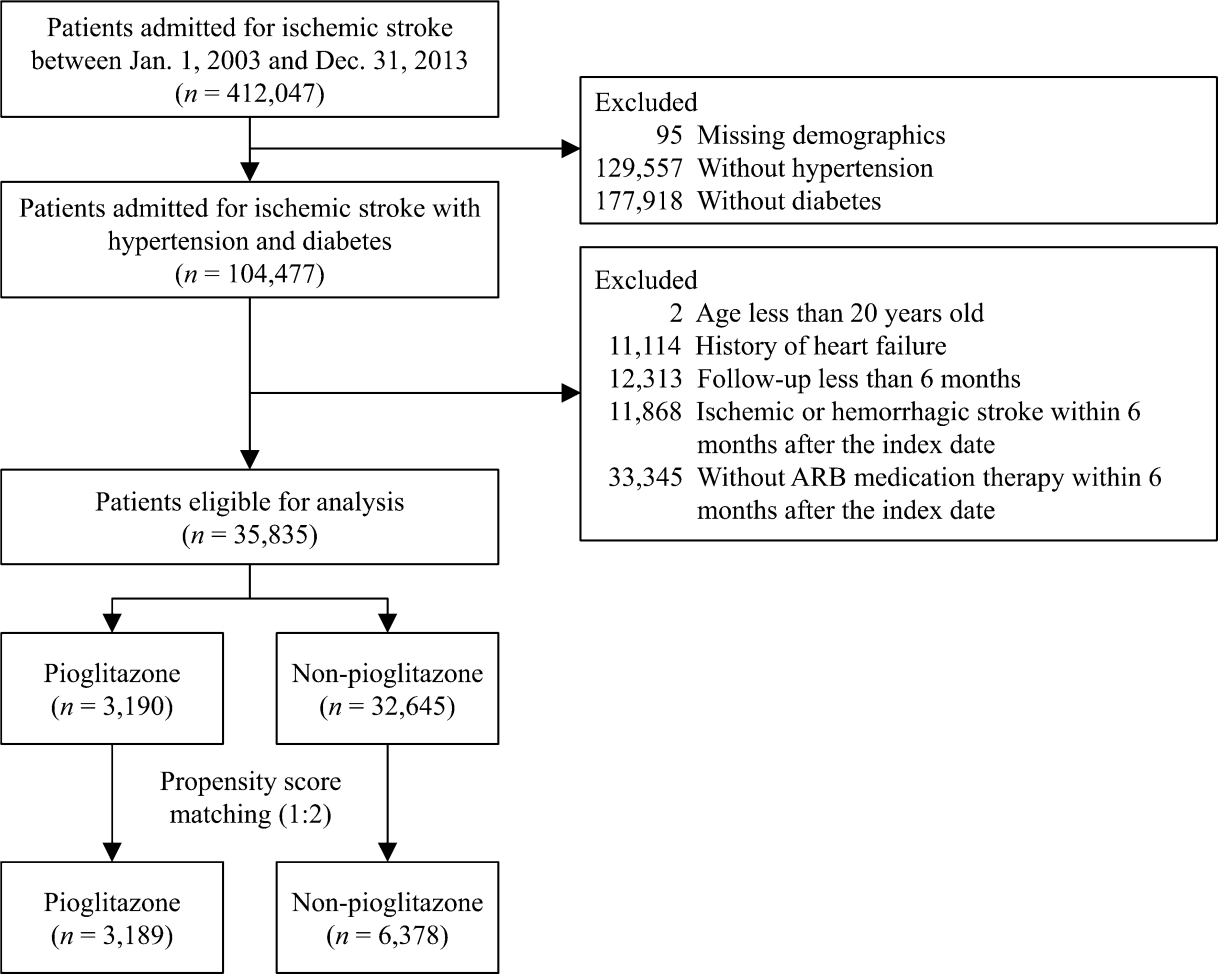
Flow chart for the inclusion of study patients. Patients with hypertension and type 2 diabetes mellitus who were hospitalized due to acute ischemic stroke were enrolled after the relevant exclusion criteria were applied. The patients were further divided into pioglitazone and non-pioglitazone groups according to their prescribed oral anti-diabetic drugs. *ARB* angiotensin receptor blocker

The present retrospective nationwide cohort study included all patients in the National Health Insurance Research Database (NHIRD), who were initially admitted to hospital due to IS between January 1st 2003 and December 31st 2013. The National Health Insurance (NHI) program covers > 99% of the population in Taiwan and the NHIRD records the data submitted to the NHI program. International Classification of Diseases, Ninth Revision, Clinical Modification (ICD-9-CM) codes are used for the registration of all diagnoses, and these data bases are routinely monitored by the NHI Bureau [20]. The patients of interest were restricted to hospitalized patients with a principal diagnosis of IS or transient ischemic attack (ICD-9-CM codes 433-435). Patients without a definite cerebral infarction were not included (ICD-9 codes of 433.00, 433.10, 433.20, 433.30, 433.80, 433.90, 434.90, 434.00, 434.10 and 434.90) [21]. Moreover, the current study focused on the pleiotropic effects of combining T2DM and HTN medications with PPAR-γ modulating properties in IS patients; those without a history of T2DM or HTN were not included. Pioglitazone may induce fluid retention and worsen heart failure (HF), patients with a history of HF were also excluded (Fig. 1). ARB based anti-hypertensive treatment was also required to minimize the class effect of different anti-hypertensive drug categories. All other exclusion criteria are shown in Fig. 1. The Ethics Institutional Review Board of Linkou Chang Gung Memorial Hospital approved the current study (Approval no. 201900714B1).

### Exposure to study drugs

The eligible patients were divided into two groups according to the oral antidiabetic agents (OADs) which were prescribed during the 6-month exposure window after the index hospitalization: (1) pioglitazone and (2) non-pioglitazone groups. In the other words, we adopted a ‘pseudo-placebo’ comparison group instead of the active comparator design. Medication was extracted from the claims data of outpatient visits or the refill for chronic illness in the pharmacy. Patients were determined to be users if the study drugs (pioglitazone or OADs) were prescribed twice (or more) in outpatient visits or once (or more) in the refill of the pharmacy. To ensure the consistent use of study drugs in each group, patients were excluded if they took any pioglitazone in the non-pioglitazone group for even 1 day during the 6-month exposure period. For the assessment of adherent medication use, we obtained the medication possession ratio (MPR) calculated by dividing the number of days of medication prescribed (numerator) by the number of days (denominator) during a time period of 6 months (183 days) after index date. The above information was extracted using the date of dispensing and supply in the claims data. Since BP and blood sugar levels were not recorded in the NHIRD, the add-on antihypertensive drugs, the average numbers of antihypertensive drugs and the types of OADs were adjusted to militate the bias associated with different levels of BP and blood sugar [22]. The index hospitalization was later defined as the first hospitalization due to IS throughout the study period.

### Ascertainment of IS, HTN and DM

The ICD-9-CM diagnostic codes of IS have been validated in two previous NHIRD studies [20, 23]. The positive predicted values of principal inpatient diagnoses were 88% in these two studies. The diagnostic codes for HTN and T2DM were also validated in a previous NHIRD study [24]. The agreement between diagnoses in the claims records and self-reports were 93% and 98% for HTM and T2DM, respectively. Besides, the agreement between relevant medications and self-reports was 87% and 95% for HTM and T2DM, respectively [24]. To avoid misclassification bias due to coding errors, the included patients had to meet both the diagnosis and medication requirements.

### Covariates

The patient’s baseline characteristics, including sex, age and hospital level during their index hospitalization, were extracted from the database. Their medical records before the index hospitalization were also obtained to track any history of comorbidities and major health events. Some patients were identified as having at least two outpatient diagnoses or an inpatient diagnosis in the previous year, including coronary artery disease, chronic kidney disease (CKD), chronic obstructive pulmonary disease, atrial fibrillation and dyslipidemia. Dialysis and malignancy were detected using the catastrophic illness certificate database. Previous stroke and myocardial infarction (MI) were detected using any inpatient diagnosis prior to the index date. Most of the diagnostic codes for these events and comorbidities were validated in previous studies (Additional file 1: Table S1) [23, 24]. Charlson Comorbidity Index scores were used to determine the patient’s overall systemic health. An estimated National Institutes of Health Stroke Scale (NIHSS) was applied to access the severity of IS; this was validated in a previous NHIRD study [25]. The use of medication including telmisartan was also captured via the Taiwan NHI reimbursement and Anatomical Therapeutic Chemical codes, which was also defined as at least two prescriptions in outpatient visits or any single refill for chronic illness in a pharmacy during the 6-month exposure window. The Anatomical Therapeutic Chemical codes used for the drugs are provided in Additional file 1: Table S2.

### Outcome measurement

With reference to previous clinical trials [8, 26, 27], the primary outcome was recurrent IS in this study. The secondary outcomes included acute MI, cardiovascular death, all-cause mortality, admission for HF, and bladder cancer. Recurrent IS was adjudicated when patients admitted primarily due to IS during the follow-up period (Principal diagnosis with ICD-9-CM codes of 433-435 except 433.00, 433.10, 433.20, 433.30, 433.80, 433.90, 434.90, 434.00, 434.10, and 434.90). MI and HF were also judged when patients who were hospitalized principally due to these diagnoses [28]. Death and causes of death were identified according to the registry data of NHIRD. The definition of all-cause mortality and cardiovascular death were the same as those in the registry data of the NHIRD [21, 22]. Bladder cancer was detected in the catastrophic illness certificate database. The follow-up period was calculated from the discharge day of the index hospitalization to the day of death, event occurrence or until December 31st 2013, whichever occurred first.

### Statistical analysis

The event rate of stroke was 6.5% and 8% in the pioglitazone and placebo groups, respectively, according to a previous randomized trial [8]. Given an alpha level of 5%, a minimum sample size of 9382 patients (4691 in each group) was required to achieve a power of 80%.

Propensity score matching (PSM) was used to balance the distribution of baseline characteristics, and the number of antihypertensive drug classes and OADs used between the two groups. The propensity score was the predicted probability of being in the pioglitazone group given the values of the selected covariates. The covariates used to calculate the propensity score were age, sex, DM duration, events and comorbidities, estimated NIHSS, antihypertensive drug class and OADs, other medications, and the index date (Additional file 1: Table S3). The greedy nearest neighbor matching algorithm was adopted and the caliper was set as 0.2 times the logit of the standard deviation of the propensity score. Replacement after matching was not allowed and the matching order was random. To minimize bias of treatment effect estimation, a 1:2 matching ratio was adopted [29]. The quality of matching was checked using the absolute standardized difference (STD) between the groups after matching, where an absolute value < 0.1 was considered have a non-substantially difference between the groups.

As for the fatal time to event outcomes (i.e. all-cause mortality and cardiovascular death), the risks between the groups were compared using the Cox proportional hazard model. The incidence of non-fatal time to event outcomes (e.g. recurrent IS) between the groups was compared using the Fine and Gray subdistribution hazard model, which considered all-cause mortality a competing risk. The study group (pioglitazone vs. non-pioglitazone) was the only explanatory variable in the survival analyses. The within-pair clustering of outcomes after propensity score matching was accounted for by using a robust standard error, which was known as a marginal model [30]. In addition to using matching as the primary analysis, a sensitivity analysis was conducted with an inverse-probability-of-treatment weighting method to examine the result of recurrent IS.

Subgroup analyses for recurrent IS were conducted on 13 pre-specified subgroup variables, including age, sex, coronary artery disease, CKD, chronic obstructive pulmonary disease, dyslipidemia, estimated NIHSS group, telmisartan, diuretics, number of anti-hypertensive agents, insulin, aspirin and clopidogrel. In an additional analysis of pioglitazone and/or telmisartan users, the linear trend of IS risk was tested across different PPAR-γ intensity groups (telmisartan only, pioglitazone only, and pioglitazone plus telmisartan) using a subdistribution hazard model. Likewise, the dose-dependent outcomes across different MPRs of pioglitazone (0%, < 80%, and ≥ 80%) on the risk of recurrent IS was also tested using a linear trend test. The two additional analyses were performed using the whole cohort with adjustments for age, sex, DM duration, all previous events and comorbidities.

Data scientists responsible for the data mining and extraction from the NHIRD were blinded to the design, grouping, and primary interest of this study. The statisticians responsible for data analyses were not blinded to the study design. However, they have received compensation and declared no competing interest between the findings of this study and their company. All statistical analyses were performed using SAS version 9.4 (SAS Institute, Cary, NC, USA), including the procedures of ‘psmatch’ for propensity score matching, ‘phreg’ for survival analysis and the macro of ‘%cif’ for generating cumulative incidence function under the Fine and Gray subdistribution hazard method. Statistical significance was set at *p *< 0.05, and no adjustment of multiple testing (multiplicity) was made during the study. The clinical significance of subgroup analyses was loosened to *p *< 0.1 because the interaction test was known to be more conservative and less powerful [31].

## Results

### Study patients

Between January 1st 2003 and December 31st 2013, a total of 412,047 patients admitted due to IS were available in the NHIRD. Totally 129,557 and 177,918 patients who did not have a history of HTN and T2DM were not included. In addition, 11,114 patients with a history of HF, 12,313 patients whose follow-up duration less than 6 months (including those who died during their index admission), and 11,868 patients who developed recurrent IS or hemorrhagic stroke within 6 months after their index hospitalization were also excluded. A total of 33,345 patients who did not receive any ARB for control of their HTN were also excluded. Finally, 35,835 IS patients were confirmed as eligible for analyses based on the inclusion/exclusion criteria. There were 3190 patients in the pioglitazone group and 32,645 patients in the non-pioglitazone group (Fig. 1).

### Baseline characteristics

Before the PSM, patients in the pioglitazone group were younger (pioglitazone vs. non-pioglitazone: 67.0 ± 10.0 vs. 68.7 ± 10.4 years old; STD = − 0.166) and had a higher prevalence of dyslipidemia (pioglitazone vs. non-pioglitazone: 52.5% vs. 43.5%; STD = 0.180), but had milder estimated NIHSS (pioglitazone vs. non-pioglitazone: 5.3 ± 3.3 vs. 5.9 ± 4.1; STD = − 0.165) and shorter follow-up durations (pioglitazone vs. non-pioglitazone: 4.0 ± 2.4 vs. 4.2 ± 2.7 years; STD = − 0.104; Additional file 1: Table S3). After PSM, all baseline characteristics and medications were well balanced between the two groups (Table 1). Besides, the duration between index IS hospitalization and the first pioglitazone exposure were 1.4 ± 1.6 months in the pioglitazone group.

**Table 1:** Characteristics of the study patients with and without use of pioglitazone after propensity score matching

Characteristics	Pioglitazone (*n* = 3189)	Non-pioglitazone (*n* = 6378)	STD
Age, years	67.0 ± 10.0	66.9 ± 10.3	0.008
Age group, n (%)			
< 65 years	1302 (40.8)	2615 (41.0)	− 0.004
65–74 years	1151 (36.1)	2336 (36.6)	− 0.011
≥ 75 years	736 (23.1)	1427 (22.4)	0.017
Male, n (%)	1580 (49.5)	3154 (49.5)	0.002
Admitted in medical center, n (%)	957 (30.0)	1995 (31.3)	− 0.028
DM duration, years	8.6 ± 3.4	8.6 ± 3.5	− 0.003
Comorbidity, n (%)			
Atrial fibrillation	89 (2.8)	154 (2.4)	0.024
Myocardial infarction	90 (2.8)	180 (2.8)	0.000
Malignancy	138 (4.3)	288 (4.5)	− 0.009
Chronic obstructive pulmonary disease	197 (6.2)	395 (6.2)	− 0.001
Chronic kidney disease	221 (6.9)	466 (7.3)	− 0.015
Dialysis	27 (0.8)	55 (0.9)	− 0.002
Old stroke	282 (8.8)	622 (9.8)	− 0.031
Coronary artery disease	787 (24.7)	1594 (25.0)	− 0.007
Dyslipidemia	1673 (52.5)	3309 (51.9)	0.012
CCI total score	3.6 ± 1.6	3.6 ± 1.6	− 0.008
Estimated NIHSS	5.3 ± 3.3	5.3 ± 3.2	0.007
Estimated NIHSS group, n (%)			
≤ 5	2480 (77.8)	4958 (77.7)	0.001
6–13	556 (17.4)	1132 (17.7)	− 0.008
> 13	153 (4.8)	288 (4.5)	0.013
Anti-hypertensive agent, n (%)			
Telmisartan	246 (7.7)	465 (7.3)	0.016
Alpha-blocker	288 (9.0)	580 (9.1)	− 0.002
Diuretics (thiazide/loop diuretics/spironolactone)	830 (26.0)	1652 (25.9)	0.003
Beta-blocker	1275 (40.0)	2630 (41.2)	− 0.026
CCB	1928 (60.5)	3835 (60.1)	0.007
Average number of anti-hypertension drugs	2.4 ± 1.1	2.5 ± 1.1	− 0.014
Antidiabetic agent, n (%)			
Insulin	560 (17.6)	1133 (17.8)	− 0.005
DPP4i	576 (18.1)	1168 (18.3)	− 0.007
Secretagogue (Glinide)	578 (18.1)	1120 (17.6)	0.015
Alpha glucosidase	828 (26.0)	1671 (26.2)	− 0.005
Biguanide (metformin)	2186 (68.5)	4327 (67.8)	0.015
Sulfonylurea	2408 (75.5)	4799 (75.2)	0.006
Other medications, n (%)			
Anticoagulant	102 (3.2)	197 (3.1)	0.006
Fibrate	444 (13.9)	907 (14.2)	− 0.009
Clopidogrel	532 (16.7)	1070 (16.8)	− 0.003
Statin	1616 (50.7)	3218 (50.5)	0.004
Aspirin	2431 (76.2)	4925 (77.2)	− 0.023
Follow-up years	4.0 ± 2.4	3.9 ± 2.4	0.022
Propensity score	0.128 ± 0.070	0.128 ± 0.070	0.002

*DM* diabetes mellitus, *CCI* Charlson Comorbidity Index, *NIHSS* National Institutes of Health Stroke Scale, *CCB* calcium channel blockers, *DPP4i* dipeptidyl peptidase-4 inhibitor, *STD* standardized difference

An absolute STD < 0.1 was considered as a non-substantially difference between the groups

### Primary outcome: recurrent ischemic stroke

**Fig. 2:**
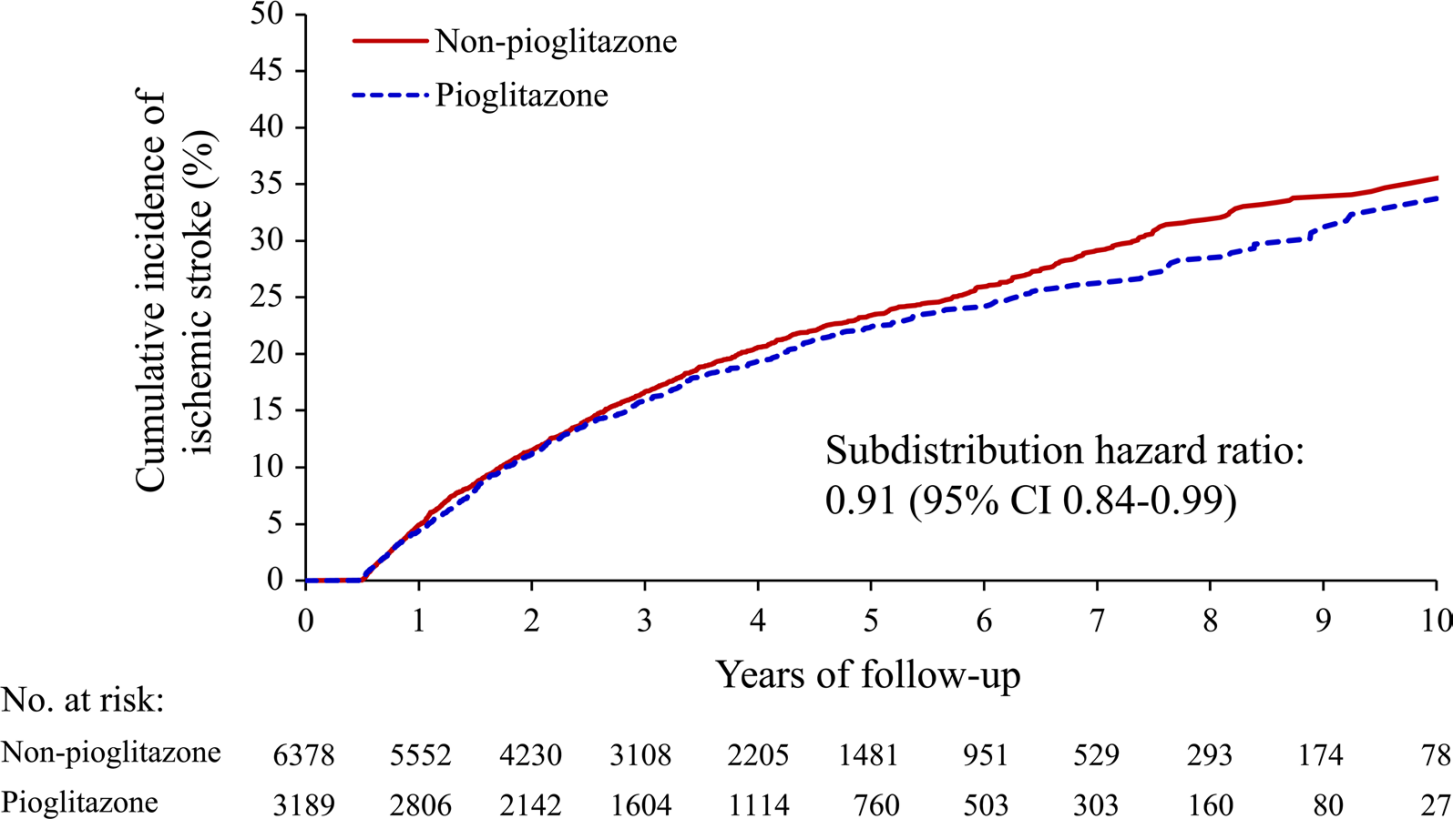
Comparison of the cumulative incidence of recurrent ischemic stroke between pioglitazone and non-pioglitazone groups in the propensity score matched cohort. The curves show lower risks of recurrent ischemic stroke in the pioglitazone group

The mean follow-up periods were similar between the pioglitazone (4.0 ± 2.4 years) and non-pioglitazone (3.9 ± 2.4 years; STD = 0.022) groups after PSM. The primary outcome was compared between the two study groups. Compared to the non-pioglitazone group, the pioglitazone group had a lower risk of recurrent IS (pioglitazone vs. non-pioglitazone: 18.8% vs. 20.0%; subdistribution hazard ratio [SHR], 0.91; 95% confidence interval [CI] 0.84–0.99; Table 2). In the sensitivity analysis using inverse-probability-of-treatment weighting, the pioglitazone group also had a lower risk of recurrent IS (pioglitazone vs. non-pioglitazone: 19.0% vs. 21.2%; SHR, 0.89; 95% CI 0.80–0.99). The cumulative incidence plot shows lower trends of recurrent IS in the pioglitazone group compared with the non-pioglitazone group (Fig. 2).

**Table 2:** Recurrent ischemic stroke and secondary safety outcomes of patients with and without use of pioglitazone

Outcome	Pioglitazone (n = 3189)	Non-pioglitazone (n = 6378)	Pioglitazone vs. non-pioglitazone	
			SHR (95% CI)	*p*-value
Primary analysis: propensity score matching				
Recurrent ischemic stroke, n (%)	598 (18.8)	1273 (20.0)	0.91 (0.84, 0.99)	0.033
Sensitivity analysis: IPTW				
Recurrent ischemic stroke, %	19.0	21.2	0.89 (0.80, 0.99)	0.025
Secondary outcomes				
Acute myocardial infarction, n (%)	119 (3.7)	265 (4.2)	0.79 (0.65, 0.97)	0.021
Hospitalization for heart failure, n (%)	200 (6.3)	410 (6.4)	0.99 (0.85, 1.15)	0.867
All-cause mortality, n (%)	560 (17.6)	1158 (18.2)	0.94 (0.83, 1.06)	0.320
Cardiovascular death, n (%)	362 (11.4)	731 (11.5)	0.95 (0.81, 1.11)	0.523
Bladder cancer, n (%)	10 (0.31)	11 (0.17)	1.34 (0.62, 2.88)	0.456

*SHR* subdistribution hazard ratio, *CI* confidence interval, *IPTW* inverse-probability-of-treatment weighting

Statistical significance was set at *p *< 0.05

### Secondary outcomes

Compared with the non-pioglitazone group, the pioglitazone group had a lower risk of acute MI (pioglitazone vs. non-pioglitazone: 3.7% vs. 4.2%; SHR, 0.79; 95% CI 0.65–0.97). The risks of all-cause mortality, cardiovascular death, admission for HF, and occurrence of bladder cancer were not significantly different between the two groups at the end of the follow-up (Table 2).

### Subgroup analyses for risks of recurrent IS

**Fig. 3:**
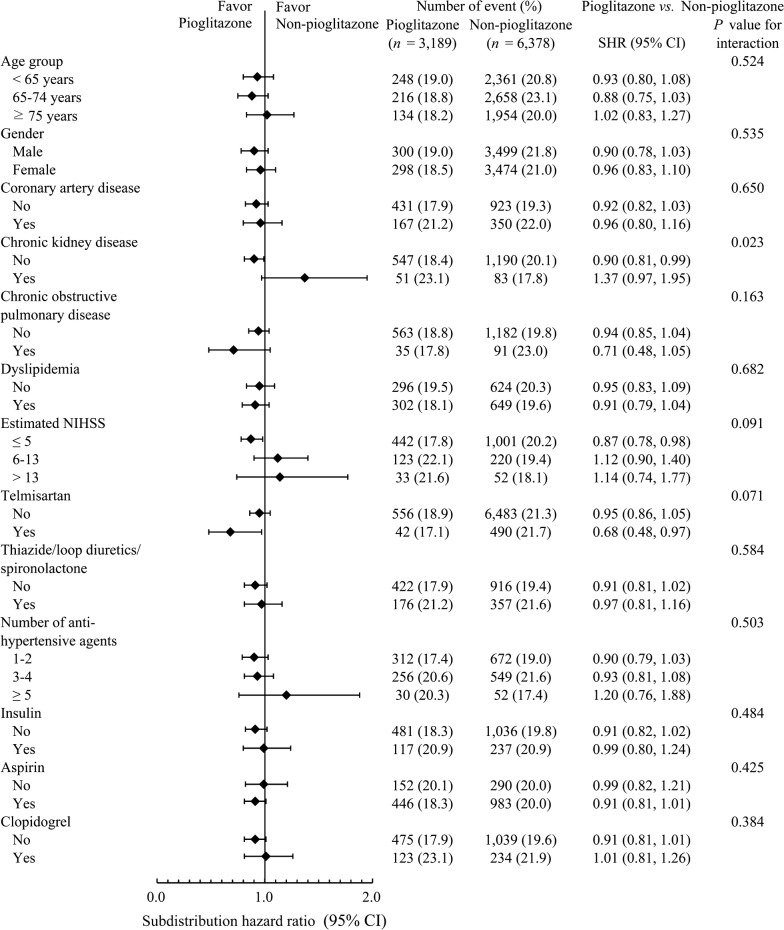
Subgroup analyses of recurrent ischemic stroke. Patients without chronic kidney disease had lower risks of recurrent IS in the pioglitazone group (*p* for interaction = 0.023). Patients using telmisartan for hypertension control may have a lower risk of recurrent IS in the pioglitazone group (*p* for interaction = 0.071). *CI* confidence interval, *SHR* subdistribution hazard ratio, *IS* ischemic stroke. Statistical significance for interaction of subgroup analyses was set at *p *< 0.1

Subgroup analyses defined by various baseline features did not disclose significant alterations to the observed effect of pioglitazone, with the exception of 2 significant interactions (*p *< 0.1; Fig. 3). The first interaction was between pioglitazone and a history of CKD before the index event. The second was between pioglitazone and concurrent telmisartan use. The observed effect of pioglitazone in reducing recurrent IS risks was less apparent in patients who had CKD or in those who used ARBs other than telmisartan for BP control.

### Additional analyses: trend test and dose-dependent effect of the intensity of PPAR-γ modulating treatments on IS

A graded correlation was observed between the intensity of PPAR-γ treatment and subsequent IS, with an overall IS rate of 21.7% for patients administered with telmisartan alone, 18.9% for patients with pioglitazone alone and 17.1% for patients with pioglitazone plus telmisartan (Table 3; *p *= 0.076 for linear trend). The dose-dependent outcome for different MPRs in the pioglitazone group also showed a borderline significant trend that higher pioglitazone adherence was associated with a lower cumulative risk of IS (unadjusted event rate: non-pioglitazone group, 21.4%; those with a MPR < 80%, 20.6%; and those with a MPR ≥ 80%, 16.4%; *p *= 0.068 for trend).

**Table 3:** Trend test and dose-dependent effect of the intensity of PPAR-γ modulating treatments on risk of recurrent ischemic stroke by using the whole cohort before matching

Trend	Patient number	No. of event (%)	*p* trend	*p* trend^‡^
Trend test of telmisartan and pioglitazone effect			0.087	0.076
Pioglitazone plus telmisartan	246	42 (17.1)		
Pioglitazone alone	2944	556 (18.9)		
Telmisartan alone	2259	490 (21.7)		
Dose-dependent effect of pioglitazone			0.015	0.068
MPR ≥ 80	1406	231 (16.4)		
MPR < 80	1784	367 (20.6)		
Non-pioglitazone	32,645	6973 (21.4)		

*PPAR-γ* peroxisome proliferator-activated receptor-gamma, *MPR* medication possession ratio

Statistical significance was set at *p *< 0.05

^‡^Adjusted for all the variables listed in Table 1, except medications, follow-up years and the propensity score

## Discussion

The current nationwide cohort study showed that IS patients taking pioglitazone for T2DM control may have lower risks of recurrent IS during long-term follow-up. Our result supports the protective effects of pioglitazone on IS recurrence in an Asian population. Previous meta-analyses have demonstrated the cardiovascular protective effects of pioglitazone; the IRIS study included non-diabetic patients with IR based on a homeostasis model assessment of insulin resistance score and demonstrated lower stroke or MI in patients with pioglitazone use than in the others receiving placebo. However, the difference in stroke risk reduction alone was not statistically significant between the two groups [8]. Although the subgroup analysis of the PROspective pioglitAzone Clinical Trial In macroVascular Events (PROactive) study showed that pioglitazone could reduce the risk of recurrent stroke in T2DM patients [26], but only 19% of the enrolled patients in this study had previous stroke and this may limit the conclusive answers of the subgroup analysis [7]. Therefore, for T2DM patients with a previous stroke, more clinical data may be needed to support the protective effects of pioglitazone on secondary prevention. Besides, these conclusions should be interpreted cautiously when applied to Asian population. In theory, Asian IS patients are more vulnerable to have recurrent IS, however the recruitment of Asian patients in these studies was insufficient. The Junteno Stroke Prevention study in Insulin Resistance and Impaired glucose Tolerance did not show a significant protective effect of pioglitazone in Japanese [27]. Nevertheless, the number of enrolled patients in that study was too small. The IRIS study also failed to demonstrate sufficient protective effects of pioglitazone in Asian patients [8, 27]. In Asian T2DM patients without prior cardiovascular diseases, the real-world data also showed controversial protective effects of pioglitazone. Chan et al. [32] demonstrated that pioglitazone added to metformin, compared to sulfonylurea plus metformin, may have fewer major cardiovascular events in T2DM patients. But another real-world study did not show the protective effects of pioglitazone on IS prevention [33]. In Asian T2DM patients with previous strokes, a Korea nested case–control study demonstrated a benefit of pioglitazone on composite cardiovascular outcomes but not on recurrent IS [12]. As a result, the strength of our nationwide cohort study showed that Asian patients with IS taking pioglitazone for T2DM could lower the risks of recurrent IS during the long-term follow-up. Our data provided the evidence of pioglitazone for secondary prevention of IS in Asian T2DM patients, which was compatible with the findings of cardiovascular protection from previous meta-analyses [7, 11, 34]. Furthermore, the current study revealed that IS patients without CKD or concurrent telmisartan use for BP control may have trends of obtaining more benefits when taking pioglitazone for T2DM.

It is well known that PPAR-γ may have metabolism regulating and neuroprotective effects and could have protective effects for HTN, atherosclerosis and CKD [5, 6, 35]. Pioglitazone, a potent PPAR-γ agonist, has demonstrated vascular protective effects in previous studies [7, 8, 11]. Pioglitazone may show potential beneficial effects on anti-inflammation, neuroprotection, and neurogenesis in cerebral ischemia animal studies [36, 37]. Moreover, pioglitazone may raise adiponectin level. The elevated adiponectin level could be associated with increased energy consumption and insulin sensitivity [38]. The elevated adiponectin might also have anti-inflammatory, anti-atherosclerotic, and anti-thrombotic effects [39]. Telmisartan may also promote the secretion of adiponectin [40]. But the correlations between adiponectin levels and clinical outcomes remain controversial and may need further investigations [41, 42]. Furthermore, it remains unknown whether the more potent PPAR-γ modulating effects may provide better vascular protective effects. Between the two clinically available thiazolidinediones, rosiglitazone has the most potent PPAR-γ activation [43]. Different from pioglitazone, rosiglitazone has an unfavorable effect on atherogenic lipid profiles with a greater elevation of low-density lipoprotein cholesterol and triglyceride, which could contribute to adverse outcomes and increase cardiovascular risks [33, 44]. Similarly, pioglitazone can increase low-density lipoprotein cholesterol over time. But it also increases high-density lipoprotein cholesterol levels, decreases triglyceride level, and has unremarkable changes in non-high-density lipoprotein cholesterol levels [45]. In our study, it was observed that the prevalence of dyslipidemia was substantially higher in the pioglitazone group than in the non-pioglitazone group before matching, therefore the patients who received pioglitazone were more likely to have a higher risk of recurrent IS. However, this effect may have been mitigated after matching because the prevalence of dyslipidemia was balanced between groups. If this indication bias actually existed, then the observed beneficial effect of pioglitazone on prevention of recurrent IS may be somewhat under-estimated in this study.

Telmisartan is apartial PPAR-γ agonist and is the only ARB that yields PPAR-γ modulating effects under clinical doses [46]. However, the PPAR-γ modulating effect of telmisartan remains much lower than that of pioglitazone [14, 46]. Therefore, telmisartan has not shown significant stroke preventive effects in previous studies [47, 48]. Telmisartan binds with the PPAR-γ receptor in a different way from pioglitazone, it is rational to use these two drugs concurrently in clinical practice [49]. Previous studies have proved the safety of combining these PPAR-γ modulating drugs [18, 19]. However, there was no clinical study designed specifically to examine the effect of combining these drugs on stroke prevention. The real-world data used in the current study, revealed that compared with those taking ARBs other than telmisartan, patients receiving telmisartan for BP control tended to have less recurrent IS (*p* for interaction = 0.071). The study further demonstrated a borderline significant trend of fewer upcoming IS events following the combined use of pioglitazone and telmisartan, compared with the use of pioglitazone or telmisartan alone (*p* for trend = 0.076). These results suggest there could be a protective effect from using more intensive PPAR-γ modulating regimens for IS patients with T2DM and HTN. Pioglitazone adherence is an important factor contributing to IS prevention [50]. Our data echoed the post hoc analysis of the IRIS study which showed the hazard ratio of recurrent IS could be lower in the patients of the subgroup with pioglitazone adherence ≥ 80% than in those of the intention-to-treat analysis [50]. A clinical trial is warranted to confirm or refute the casual relationship of such combination therapy.

Chronic kidney disease is an independent risk factor for carotid atherosclerosis and stroke [51], which might be related to vascular calcification, endothelial dysfunction, and impaired drug effects for secondary stroke prevention in these patients [51]. IR is also a cardiovascular risk factor in patients with CKD [52]. Pioglitazone activates PPAR-γ and hence may improve IR in patients with CKD [53]. The vascular protective effect of pioglitazone in CKD patients was unknown in the IRIS study [53]. However, data from the PROactive study demonstrated that CKD patients treated with pioglitazone for T2DM may have higher all-cause death, MI and stroke when compared to non-CKD patients [7, 54]. There is very limited real-world data comparing pioglitazone use between CKD and non-CKD patients. Similar to the PROactive trial, our study also showed that non-CKD patients may have more benefits on IS prevention from pioglitazone use (*p* for interaction = 0.023). At low doses, when compared to standard doses, pioglitazone is associated with comparable glycemic control but with reduced weight gain and fluid retention in CKD patients [55]. The drug adherence of pioglitazone could be lower in CKD patients in clinical practice because CKD is a major predictor of hospitalization for HF [56]. However, our data showed both the MPR (CKD vs. non-CKD: 67.3 ± 31.8% vs. 64.0 ± 33.8%; *p *= 0.157) and mean doses (CKD vs. non-CKD: 18.3 ± 11.1 vs. 18.2 ± 12.3 mg/day; *p *= 0.932) of pioglitazone were similar between the CKD and non-CKD patients of the pioglitazone group. Our results suggest the decreased protective effect of pioglitazone in CKD patients may come from the cardiovascular risks of renal disease itself rather than the insufficient drug compliance in these patients.

In line with previous reports, the current data also showed a lower incidence of MI in pioglitazone users [57]. Although the potential risks of HF and bladder cancer in pioglitazone users have gained clinical attention [58, 59], the issue of increasing risks of bladder cancer remains unconfirmed due to the conflicting results from previous studies [58–60]. In the present study, using the real-world data in Taiwan, the increased risk of newly diagnosed bladder cancer in the pioglitazone group was substantial by 34% (HR = 1.34) but not statistically significant due to the limited sample size. Although whether pioglitazone could lead to an increased risk of bladder cancer is with doubtful evidences, Food and Drug Administration in the United States announced that pioglitazone should not be used in patients with active bladder cancer [61]. Therefore, in patients with a history of bladder cancer, the benefits of pioglitazone, such as stroke prevention versus the possible risks of cancer recurrence should be considered carefully and pioglitazone is probably under-utilized in our real-world practice [62].

There were some limitations to the present study. First, the homeostasis model assessment of the insulin resistance index, blood sugar and BP levels were not available in the claims database. The stroke severity of the included patients was also not known. Besides, patients who developed recurrent minor IS without hospitalization were not registered in this data. Patients were followed after a 6-month exposure window of the OADs, therefore high-risk patients who were vulnerable to develop recurrent IS within 6 months after index hospitalization were excluded in this study. All of these may lead to selection bias of the study population and data interpretation. This study was also unable to demonstrate the short-term effect of pioglitazone and intensive PPAR-γ modulating treatment on risks of recurrent IS during the 6-month follow-up after the index IS, and a clinical trial in the future is warranted to answer this question. However, some efforts were made to mitigate these selection biases, residual or unmeasured confounders. The estimated NIHSS, the number of antihypertensive drug agents and antidiabetic drugs used at baseline, and the Charlson Comorbidity Index were used to be proxies of stroke severity index, BP control and glucose control, respectively. Second, drug switching, combinations, and adherence may confound the results. In the real-world study, we can only speculate the drug adherence and compliance through prescription records. Unlike clinical trials, we can hardly obtain the actual medication-taking behavior of patients and the drug prescription behavior of physicians in claims data. However, all the insurance claims are reviewed and inspected by medical reimbursement specialists in Taiwan. Physicians and their institutions are penalized if they violate clinical guidelines. In addition, we examined the association between different MPRs and IS events in the present study. This may reduce the bias that might influence the conclusions. Third, the statistical analysis may be underpowered due to limited sample size, even though this have been a population-based study. For some physicians, ARBs may not be their drug of choices for hypertensive control in IS patients. However, this national cohort data could be the largest available one we can achieve to answer this unknown question so far. Fourth, the ICD-9-CM may have been coded incorrectly in the claims database. However, validation studies have been conducted previously by linking this coding method and the stroke registry. Fifth, the causal effects of these study drugs should be interpreted cautiously in this observational study. Population-based observational studies support a lower standard of evidence than randomized control trials. The exploratory results of our observational study remained insufficient to give conclusive answers. However, our findings could help to motivate future studies on more potent PPAR-γ modulating treatment in IS patients. Lastly, the generalizability of these conclusions to other ethnicities is unclear.

## Conclusion

The current study suggests that the use of pioglitazone in IS patients with T2DM and HTN is associated with fewer recurrences of IS in an Asian population. Those who have concurrent telmisartan use or a higher MPR may have a trend of more pleiotropic effects, which might be associated with higher PPAR-γ effects. Clinical trials and basic researches should be conducted to confirm or refute the potential effects of more intensive PPAR-γ-modulating treatments upon clinical outcomes in hypertensive T2DM patients after IS and the possible mechanism.

## Supplementary information

**Additional file 1: Table S1.** ICD-9-CM code used for diagnosis in the current study. **Table S2.** Anatomical Therapeutic Chemical (ATC) codes used for drugs in the current study. **Table S3.** Characteristics of the study patients with and without use of pioglitazone before propensity score matching.

## Data Availability

The datasets used and analyzed in our study are available from the corresponding author on reasonable requests.

## Abbreviations

PPAR-γ: peroxisome proliferator-activated receptor-γ
T2DM: type 2 diabetes mellitus
IS: ischemic stroke
IR: insulin resistance
OADs: oral antidiabetic agents
HTN: hypertension
ARBs: angiotensin receptor blockers
BP: blood pressure
CKD: chronic kidney disease
MPR: medication possession ratio
MI: myocardial infarction
HF: heart failure
NHIRD: National Health Insurance Research Database
NIHSS: National Institutes of Health Stroke Scale
PSM: propensity score matching
STD: standardized difference
SHR: subdistribution hazard ratio
CI: confidence interval
