# Supplementary material for: Pioglitazone and PPAR-γ modulating treatment in hypertensive and type 2 diabetic patients after ischemic stroke: a national cohort study
Source: Cardiovasc Diabetol. 2020 Jan 7;19:2. doi: 10.1186/s12933-019-0979-x (PMC6945719; doi:10.1186/s12933-019-0979-x)
Supplement: Supplementary file 1 — Additional file 1: Table S1. ICD-9-CM code used for diagnosis in the current study. Table S2. Anatomical Therapeutic Chemical (ATC) codes used for drugs in the current study. Table S3. Characteristics of the study patients with and without use of pioglitazone before propensity score matching. [file 12933_2019_979_MOESM1_ESM.docx]

**Additional Table S1.** ICD-9-CM code used for diagnosis in the current study

| Variable | Code |
| --- | --- |
| Ischemic stroke | 433.xx–435.xx, excluding 433.00, 433.10, 433.20, 433.30, 433.80, 433.90, 434.00, 434.10, 434.90 |
| Hypertension | 401.xx–405.xx and any antihypertension drugs |
| Diabetes mellitus | 250.xx and any oral hypoglycemic drugs and insulin |
| Heart failure | 428.xx |
| Any stroke | 430.xx–437.xx |
| Previous myocardial infarction | 410.xx, 412.xx |
| Coronary artery disease | 410.xx–414.xx |
| Chronic kidney disease | 580.xx–589.xx, 403.xx–404.xx, 016.0x, 095.4x, 236.9x, 250.4x, 274.1x, 442.1x, 447.3x, 440.1x, 572.4x, 642.1x, 646.2x, 753.1x, 283.11, 403.01, 404.02, 446.21 |
| Dialysis | 585.xx (Catastrophic illness certificate) |
| Chronic obstructive pulmonary disease | 491.xx, 492.xx, 496.xx |
| Atrial fibrillation | 427.31 |
| Dyslipidemia | 272.xx and any lipid-lowing agents |
| Malignancy | 140.xx–208.xx (Catastrophic illness certificate) |
| Cirrhosis | 571.2, 571.5, 571.6 |
| Cardiovascular death | 390.xx–459.xx, 785.5x |
| Acute myocardial infarction | 410.xx |
| Bladder cancer | 188.xx (Catastrophic illness certificate) |

ICD-9-CM, International Classification of Diseases, Ninth Revision, Clinical Modification.

**Additional Table S2.** Anatomical Therapeutic Chemical (ATC) codes used for drugs in the current study

| Medications | ATC code |
| --- | --- |
| **Study drugs** |  |
| ARB | C09 |
| Pioglitazone | A10BG03, A10BD05 |
| **Antihypertensive drugs** |  |
| Telmisartan | C09CA07, C09DB04, C09DA07 |
| Alpha-blocker | C02 |
| Diuretics(Thiazide/Loop diuretics/Spironolactone) | C03AA03, 03AA06, C03AA91, C03AA07, C03CA01, C03CA02, C03DA01, C03EA01 |
| Beta-blocker | C07 |
| CCB | C08 |
| **Antidiabetic drugs** |  |
| Insulin | A10A |
| DPP4i | A10BH |
| Secretagogue (Glinide) | A10BX02, A10BX03 |
| Alpha glucosidase | A10BF |
| Biguanide (Metformin) | A10BA |
| Sulfonylurea | A10BB |
| **Other medications** |  |
| Anticoagulant | B01AA02, B01AA03, B01AE07, B01AF01, B01AF02, B01AF03 |
| Fibrate | C10AB01, C10AB02, C10AB03, C10AB04, C10AB05, C10AB06, C10AB09 |
| Clopidogrel | B01AC04, B01AC30 |
| Statin | C10AA |
| Aspirin | B01AC06 |

ARB, angiotensin II receptor blockers; CCB, calcium channel blockers; DPP4i,dipeptidyl peptidase-4 inhibitor.

**Additional Table S3.** Characteristics of the study patients with and without use of pioglitazone before propensity score matching

| Characteristics | Pioglitazone  (*n* = 3,190) | Non-pioglitazone  (*n* = 32,645) | STD |
| --- | --- | --- | --- |
| Age, years | 67.0±10.0 | 68.7±10.4 | -0.166 |
| Age group, n (%) |  |  |  |
| <65years | 1,303 (40.8) | 11,325 (34.7) | 0.127 |
| 65-74years | 1,151 (36.1) | 11,530 (35.3) | 0.016 |
| ≥75years | 736 (23.1) | 9,790 (30.0) | -0.157 |
| Male, n (%) | 1,581 (49.6) | 16,069 (49.2) | 0.007 |
| Admitted in medical center, n (%) | 957 (30.0) | 10,423 (31.9) | -0.042 |
| DM duration, years | 8.6±3.4 | 7.2±3.8 | 0.379 |
| Comorbidity, n (%) |  |  |  |
| Atrial fibrillation | 89 (2.8) | 1,532 (4.7) | -0.100 |
| Myocardial infarction | 90 (2.8) | 1,060 (3.2) | -0.025 |
| Malignancy | 138 (4.3) | 1,652 (5.1) | -0.035 |
| Chronic obstructive pulmonary disease | 197 (6.2) | 2,583 (7.9) | -0.068 |
| Chronic kidney disease | 222 (7.0) | 2,229 (6.8) | 0.005 |
| Dialysis | 27 (0.8) | 504 (1.5) | -0.064 |
| Old Stroke | 282 (8.8) | 3,439 (10.5) | -0.057 |
| Coronary artery disease | 787 (24.7) | 8,389 (25.7) | -0.024 |
| Dyslipidemia | 1,674 (52.5) | 14,214 (43.5) | 0.180 |
| CCI total score | 3.6±1.6 | 3.6±1.6 | 0.005 |
| Estimated NIHSS | 5.3±3.3 | 5.9±4.1 | -0.165 |
| Estimated NIHSS group, n (%) |  |  |  |
| ≤5 | 2,481 (77.8) | 23,676 (72.5) | 0.122 |
| 6-13 | 556 (17.4) | 6,462 (19.8) | -0.061 |
| >13 | 153 (4.8) | 2,507 (7.7) | -0.119 |
| Anti-hypertensive agent, n (%) |  |  |  |
| Telmisartan | 246 (7.7) | 2,259 (6.9) | 0.030 |
| Alpha-blocker | 288 (9.0) | 3,452 (10.6) | -0.052 |
| Diuretics(Thiazide/Loop diuretics/Spironolactone) | 831 (26.1) | 7,955 (24.4) | 0.039 |
| Beta-blocker | 1,276 (40.0) | 13,213 (40.5) | -0.010 |
| CCB | 1,929 (60.5) | 20,395 (62.5) | -0.041 |
| Average number of anti-hypertension drugs | 2.4±1.1 | 2.5±1.1 | -0.025 |
| Antidiabetic agent, n (%) |  |  |  |
| Insulin | 560 (17.6) | 5,918 (18.1) | -0.015 |
| DPP4i | 577 (18.1) | 3,794 (11.6) | 0.183 |
| Secretagogue (Glinide) | 579 (18.2) | 4,209 (12.9) | 0.146 |
| Alpha glucosidase | 829 (26.0) | 5,182 (15.9) | 0.251 |
| Biguanide (Metformin) | 2,186 (68.5) | 20,299 (62.2) | 0.134 |
| Sulfonylurea | 2,409 (75.5) | 20,329 (62.3) | 0.289 |
| Other medications, n (%) |  |  |  |
| Anticoagulant | 102 (3.2) | 1,817 (5.6) | -0.116 |
| Fibrate | 445 (13.9) | 3,709 (11.4) | 0.078 |
| Clopidogrel | 532 (16.7) | 5,628 (17.2) | -0.015 |
| Statin | 1,617 (50.7) | 13,304 (40.8) | 0.200 |
| Aspirin | 2,432 (76.2) | 24,187 (74.1) | 0.050 |
| Follow-up years | 4.0±2.4 | 4.2±2.7 | -0.104 |
| Propensity score | 0.128±0.070 | 0.085±0.057 | 0.666 |

DM, diabetes mellitus; CCI, Charlson Comorbidity Index; NIHSS, National Institutes of Health Stroke Scale; CCB, calcium channel blockers; DPP4i, dipeptidyl peptidase-4 inhibitor; STD, standardized difference.

An absolute STD <0.1 was considered as a non-substantially difference between the groups.
